# Meibomian gland changes in breast cancer patients treated with docetaxel-partial results

**DOI:** 10.22336/rjo.2023.21

**Published:** 2023

**Authors:** Elena Andreea Stoicescu, Alina Popa Cherecheanu

**Affiliations:** * Faculty of Medicine, „Carol Davila” University of Medicine and Pharmacy, Bucharest, Romania; Department of Ophthalmology, University Emergency Hospital, Bucharest, Romania

**Keywords:** epiphora, breast cancer, Meibomian glands, docetaxel

## Abstract

**Aim:** The purpose of the present study was to demonstrate that the narrowing and/ or atrophy of the Meibomian glands is the cause of the occurrence of hyperlacrimation in women who suffer from breast cancer and who have docetaxel in their treatment regimen.

**Method:** The study involved 10 patients diagnosed with breast cancer, who received docetaxel as treatment (study group), and 10 breast cancer patients receiving other chemotherapy treatment (control group). The study was a prospective, controlled and comparative. We mainly analyzed two very important indicators, non-invasive tear film breaking time (NKBUT) and meibography.

**Results:** A decrease and/ or narrowing of Meibomian glands in the study group (breast cancer patients treated with docetaxel) was observed on the meibography. Also, a decrease of the NKBUT was observed in the study group. The average variation of NKBUT in docetaxel patients (22%) and the average variation of meiboscopy in docetaxel patients (33%) showed the effect of docetaxel over time compared to patients who received other anticancer therapy, in whom the mean variation was very small, natural.

**Conclusions:** The action of docetaxel at the level of the two studied indicators (NKBUT and Meiboscopy) was noteworthy at the level of the study group, the changes observed in the Meibomian glands being reversible. They resolved within a few weeks of completion of docetaxel treatment.

**Abbreviations: **RE = right eye, OSD = ocular surface disease, NKBUT = noninvasive keratography tear breaking time

## Introduction

The lacrimal apparatus consists of glands that secrete tears, which are eliminated from the gland by a system of tubules and ducts [**1**,**2**]. Various ocular surface diseases can occur when an imbalance in the tear film, eyelids or lacrimal glands is observed.

Diseases of the ocular surface (OSD) were first described by John Dart, who described several conditions with different pathogenic mechanisms that produce an imbalance in the ocular surface [**3**].

Excessive lacrimation or epiphora is mainly caused by increased secretion of the lacrimal glands or difficulty in draining tears normally through the nasolacrimal ducts. In breast cancer patients receiving antineoplastic treatment with docetaxel, epiphora (excessive lacrimation) is one of the most common ophthalmic adverse reactions [**4**].

This antineoplastic, docetaxel, belongs to the class of taxoids. It is used as monotherapy or alongside other chemotherapy [**5**]. Once it was used as a treatment in breast cancer patients, their life expectancy increased [**6**]. The therapeutic indications for docetaxel are breast cancer monotherapy or in combination with other chemotherapy [**6**,**7**], hormone-resistant or metastasized prostate cancer (with other chemotherapy) [**8**], non-small cell lung cancer [**9**,**10**], gastric adenocarcinoma [**11**] and head and neck cancer [**12**]. Like other antineoplastic drugs, docetaxel also presents other adverse reactions, namely hematological reactions (febrile neutropenia, etc.) [**13**], cardiac reactions (tachycardias, heart failure, etc.) [**14**], gastrointestinal reactions [**15**], hypersensitivity reactions [**16**], nervous system disorders (sensory and motor neuropathies) [**17**], respiratory reactions (respiratory distress syndrome, etc.) [**18**].

It is not yet known what is the exact cause of hyperlacrimation in breast cancer patients taking docetaxel. An obstruction and/ or stenosis of the tear apparatus was considered anywhere along its route, but without an obvious statistical demonstration [**19**-**21**]. However, there are sufficient studies to show that epiphora is more common in women who take docetaxel weekly than in those who take it every three-weeks [**22**,**23**].

## Methods

This study was conducted on 10 breast cancer patients receiving docetaxel treatment (study group) and 10 breast cancer patients receiving other chemotherapy treatment (control group). The study was prospective, controlled and comparative. The entire research was carried out in the Ophthalmology Department, University Emergency Hospital Bucharest. The inclusion criteria were the following: patients older than 18 years receiving docetaxel treatment for breast cancer and patients receiving another chemotherapy drug for breast cancer, with a signed informed consent before performing procedures related to the study, patients being able to understand and comply with the requested procedures. The exclusion criteria were the following: history of severe damage to the ocular surface before starting chemotherapy treatment (for both groups of patients), patients who had ophthalmic surgery in the last 3 months preceding the study (for both groups of patients), patients with other eye diseases, such as glaucoma and/ or any other eye involvement that threatened visual acuity (for both groups of patients). Patients in this study had five eye examinations, namely prior to the initiation of docetaxel therapy (t0), at 1 month (t1), at 3 months (t3), at 6 months (t6) and 2 months after stopping docetaxel (t8). We mainly analyzed two very important indicators for the ocular surface, namely NKBUT and meibography (using Ocular Keratograph 5M-OCULUS, SN 7700034648130).

## Results

**Fig. 1:**
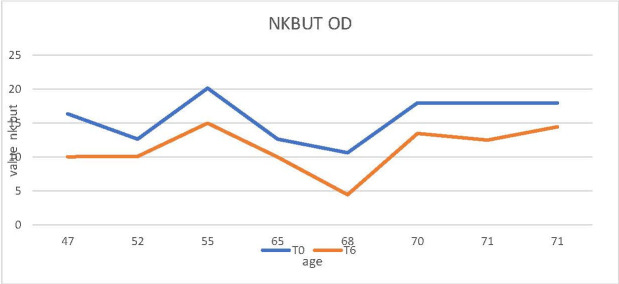
Decrease in NKBUT from time t0 to time t6 in patients receiving docetaxel chemotherapy

**Fig. 2:**
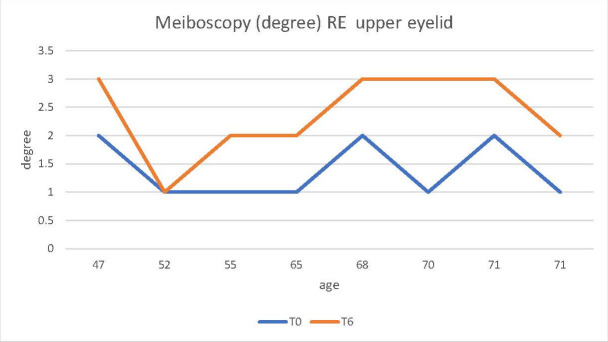
Increased narrowing and/ or decreased number of Meibomian - meibography glands in patients starting docetaxel treatment from time t0 to time t6

The NKBUT and Meibography were analyzed in the upper eyelid RE at t0 - before docetaxel was administered for the study group and before the start of the chemotherapy treatment with another neoplastic for the control group. Decreased NKBUT was observed at time t6 compared to time t0 (t6 represented the patient’s consultation number four - 6 months after the start of treatment with docetaxel or with another chemotherapy drug) (**Fig. 1**,**2**).

**Fig. 3:**
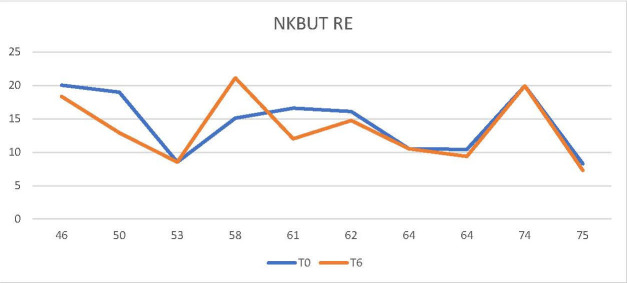
The NKBUT did not register changes from time t0 to t6 (control group)

**Fig. 4:**
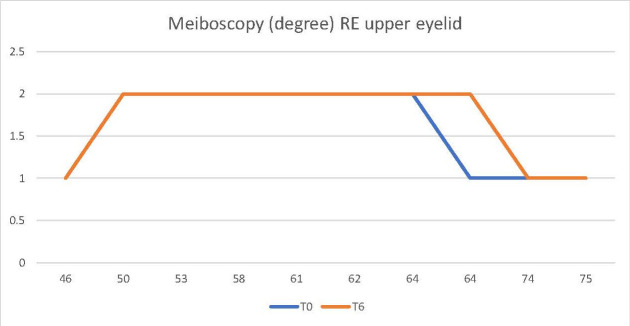
Meiboscopy did not undergo changes during the 6 months of antineoplastic treatment, other than docetaxel (control group)

For the control group of patients undergoing treatment with another antineoplastic, the two indicators (NKBUT and Meibography/ Meiboscopy) remained invariable during the 6 months of treatment, as they did not support considerable changes (**Fig. 3**,**4**).

**Fig. 5:**
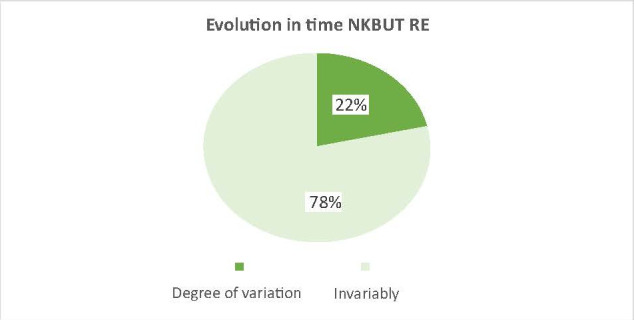
The average variation of the NKBUT on the study group, between t0 and t6 (22% average variation in patients receiving docetaxel with a decrease in NKBUT)

**Fig. 6:**
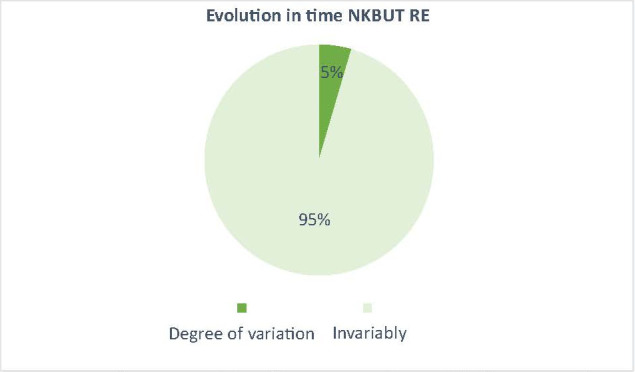
The average variation of the NKBUT on the control lot, between t0 and t6 (5% average variation of patients with other chemotherapy treatments had a decrease in NKBUT, a very small value, natural average value)

**Fig. 7:**
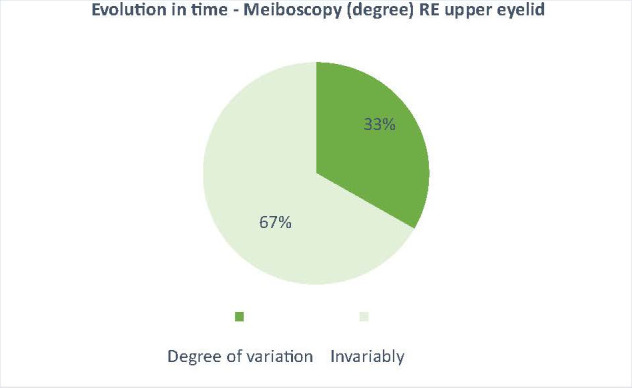
The average variation of the degree of meiboscopy on the study group, between t0 and t6 (33% average variation in patients receiving docetaxel, who showed changes on meiboscopy - a narrowing and/ or atrophy of the Meibomian glands)

**Fig. 8:**
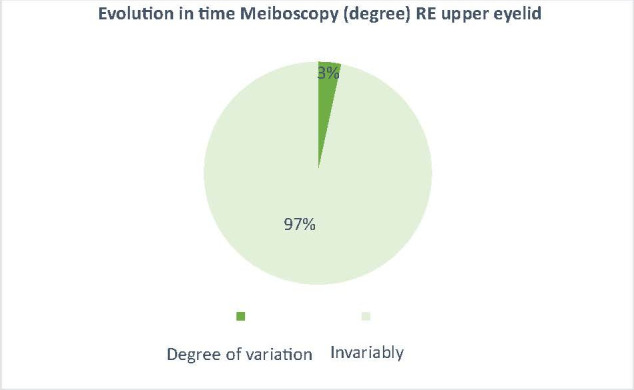
The average variation of the degree of meiboscopy on the control group, between t0 and t6 (3% average variation in patients undergoing other chemotherapy treatment with changes on meiboscopy)

The effect of the drug (docetaxel) over time was also observed by the mean variation of NKBUT and meiboscopy in patients treated with docetaxel compared to patients treated with other anticancer drugs, where the variation was very small (3% for meiboscopy and 5% for NKBUT - **Fig. 5**-**8**).

Thus, it could be observed that in the control group, treated with an antineoplastic drug other than docetaxel, the two parameters, meiboscopy and NKBUT, did not undergo considerable changes during the 6 months of treatment (**Fig. 3**,**4**). In contrast, in the study group patients receiving docetaxel, an increase in the degree of narrowing and/ or decrease in the number of Meibomian glands was observed on the meibography, during the treatment period (**Fig. 2**). Likewise, a decrease in NKBUT could be observed in these patients (**Fig. 1**).

## Discussion

Through this study we tried to demonstrate that epiphora occurs in breast cancer patients receiving docetaxel as chemotherapy, the cause being the production of morphological changes in the Meibomian glands. The changes were reversible upon completion of treatment, with epiphora disappearing within a few weeks of completion of docetaxel treatment in most patients.

There are three randomized phase III trials demonstrating an improvement in life expectancy once docetaxel was introduced into the regimen compared to patients treated with anthracyclines [**24**-**26**]. In cancer, the docetaxel regimen is administered intravenous every three weeks (20-33.3 mg/ m2/ week) [**27**]. A good systemic tolerability with weekly administration of docetaxel is 30-36 mg/ m2 [**28**]. There are studies showing that hyperlacrimation occurs more frequently when docetaxel is administered weekly than when administered every three weeks [**29**]. For example, Burstein et al. demonstrated in one study that excessive lacrimation occurs in half of women who were treated with docetaxel [**30**]. Epiphora usually occurs 12-16 weeks after starting docetaxel treatment [**30**]. It has been assumed that canalicular stenosis is the cause of epiphora, but this has not been statistically significant [**29**,**31**]. Patients with moderate canal stenosis received a lower cumulative dose of docetaxel than those with severe canal stenosis, but the difference was not statistically significant [**31**,**32**]. Some authors suggest implanting silicone tubes into the tubules temporarily to resolve excessive tearing in patients receiving docetaxel weekly early in treatment [**33**,**34**]. In patients receiving docetaxel every three weeks, some authors indicate the consult every six weeks, instead of probe and irrigation of the lacrimal system and a short course of topical corticosteroid [**35**]. Epiphora is reversible, this being supported by studies showing that 70% of patients no longer have it 4 months after the completion of treatment, 29% have an intermittent epiphora and 1% still accuse its presence [**36**].

## Conclusion

Epiphora is the most commonly reported ophthalmologic adverse reaction by women diagnosed with breast cancer and receiving treatment with docetaxel. It is more common in women treated weekly than in women treated with docetaxel every three weeks. Epiphora is self-limited, disappearing a few weeks after completing docetaxel treatment, but when present, it decreases the quality of life. 

Because epiphora also occurs in women who do not have canalicular stenosis, it cannot be stated that canalicular stenosis is the cause of epiphora.

The aim of this paper was to highlight why excessive lacrimation that occurs in women with breast cancer treated with docetaxel narrows and/ or decreases in the number of Meibomian glands. In the results presented above for the 2 groups of patients, the 2 studied parameters (NKBUT and meiboscopy) underwent changes during the treatment period in the study group (patients undergoing treatment with docetaxel). The results showed a decrease in NKBUT and an increase in the degree of narrowing and/ or reduction of the number of Meibomian glands in patients who started treatment with docetaxel from t0 to t6 (**Fig. 1**,**2**). Also, the evolution over time of these 2 parameters (NKBUT and meiboscopy) was visible, 22% of the patients suffering a decrease in NKBUT during the docetaxel treatment, compared to 5% of the patients undergoing other treatment, a percentage that represented a very small average variation. Similarly, for meiboscopy, 33% presented an average variation of the parameter in patients receiving docetaxel compared to 3%, the average variation in patients undergoing another treatment. So, it can be concluded that the effect of docetaxel was visible in the study group, treated with docetaxel.

Future prospective studies are likely to support this hypothesis, that changes in the structure and number of meibomian glands are the cause of epiphora in patients receiving docetaxel, changes that are reversible several weeks after the treatment ends.

**Conflict of Interest statement**

The authors state no conflict of interest. 

**Informed Consent and Human and Animal Rights statement**

Informed consent has been obtained from all individuals included in this study. 

**Authorization for the use of human subjects**

Ethical approval: The research related to human use complies with all the relevant national regulations, institutional policies, is in accordance with the tenets of the Helsinki Declaration, and has been approved by the Ethics Committee of the University Emergency Hospital, Bucharest, Romania.

**Acknowledgements**

None.

**Sources of Funding**

None.

**Disclosures**

None.
